# Effect of Bacterial Infection on Ghrelin Receptor Regulation in Periodontal Cells and Tissues

**DOI:** 10.3390/ijms23063039

**Published:** 2022-03-11

**Authors:** Andressa V. B. Nogueira, Marjan Nokhbehsaim, Anna Damanaki, Sigrun Eick, Svenja Beisel-Memmert, Christian Kirschneck, Agnes Schröder, Thamiris Cirelli, Natalia D. P. Leguizamón, Joni A. Cirelli, James Deschner

**Affiliations:** 1 Department of Periodontology and Operative Dentistry, University Medical Center of the Johannes Gutenberg University, 55131 Mainz, Germany; adamanak@uni-mainz.de (A.D.); james.deschner@uni-mainz.de (J.D.); 2 Section of Experimental Dento-Maxillo-Facial Medicine, Center of Dento-Maxillo-Facial Medicine, University of Bonn, 53111 Bonn, Germany; m.saim@uni-bonn.de; 3 Laboratory of Oral Microbiology, Department of Periodontology, University of Bern, 3010 Bern, Switzerland; sigrun.eick@zmk.unibe.ch; 4 Department of Orthodontics, Center of Dento-Maxillo-Facial Medicine, University of Bonn, 53111 Bonn, Germany; svenja.memmert@ukbonn.de; 5 Department of Orthodontics, University Medical Centre of Regensburg, 93053 Regensburg, Germany; christian.kirschneck@klinik.uni-regensburg.de (C.K.); agnes.schroeder@ukr.de (A.S.); 6 Department of Diagnosis and Surgery, School of Dentistry at Araraquara, São Paulo State University-UNESP, Araraquara 14801-385, SP, Brazil; thamiriscirelli@hotmail.com.br (T.C.); natidaponte@gmail.com (N.D.P.L.); joni.cirelli@unesp.br (J.A.C.)

**Keywords:** ghrelin receptor, human gingival fibroblast, *Fusobacterium nucleatum*, periodontitis, periodontal tissues

## Abstract

The effect of bacterial infection on the expression of growth hormone secretagogue receptor (GHS-R) was investigated in periodontal cells and tissues, and the actions of ghrelin were evaluated. GHS-R was assessed in periodontal tissues of rats with and without periodontitis. Human gingival fibroblasts (HGFs) were exposed to *Fusobacterium nucleatum* in the presence and absence of ghrelin. GHS-R expression was determined by real-time PCR and immunocytochemistry. Furthermore, wound healing, cell viability, proliferation, and migration were evaluated. GHS-R expression was significantly higher at periodontitis sites as compared to healthy sites in rat tissues. *F. nucleatum* significantly increased the GHS-R expression and protein level in HGFs. Moreover, ghrelin significantly abrogated the stimulatory effects of *F. nucleatum* on CCL2 and IL-6 expressions in HGFs and did not affect cell viability and proliferation significantly. Ghrelin stimulated while *F. nucleatum* decreased wound closure, probably due to reduced cell migration. Our results show original evidence that bacterial infection upregulates GHS-R in rat periodontal tissues and HGFs. Moreover, our study shows that ghrelin inhibited the proinflammatory actions of *F. nucleatum* on HGFs without interfering with cell viability and proliferation, suggesting that ghrelin and its receptor may act as a protective molecule during bacterial infection on periodontal cells.

## 1. Introduction

Lately, periodontal disease has been considered as one of the most prevalent human pathologies worldwide [1]. Periodontitis is a chronic inflammatory disease with a serious impact on different aspects of an individual’s life, leading to masticatory dysfunction and oral disability, impaired quality of life, social problems, and a significant economic burden [2,3,4]. It is a multifactorial disease characterized by the loss of tooth-supporting tissues such as gingiva, periodontal ligament, cementum, and alveolar bone, and it is associated with oral biofilm dysbiosis [2]. After bacterial challenge, a complex interaction between periodontopathogens and host immunity arises so that host resident and infiltrating cells are activated. These cells produce various proinflammatory mediators, including interleukin (IL)-1β, IL-6, IL-8, tumor necrosis factor (TNF)-α, cyclooxygenase-2 (COX2), and chemokine CC motif ligand 2 (CCL2), as well as matrix-degrading proteases such as matrix metalloproteinases [5,6,7]. If the host immune response is insufficient to remove the microbial challenge, it may cause chronic inflammation, which leads to irreversible degradation of the extracellular matrix and resorption of the alveolar bone, thus resulting in periodontal breakdown and even tooth loss.

Intrinsic and extrinsic factors consisting of genetic predisposition, systemic diseases, smoking, and stress may contribute to the onset and progression of periodontitis [8]. Various meta-analyses have suggested an association between periodontitis and systemic diseases, such as type 2 diabetes, cardiovascular disease, obesity, and metabolic syndrome [9,10,11,12,13]. Interestingly, increased body weight is associated with a higher incidence of periodontitis [14]. As a result of this interaction between body weight and periodontitis, ghrelin has become a focus of interest and has been considered as a key molecule linking periodontitis to obesity. Ghrelin has an essential role in many biological processes such as appetite level regulation, the body’s energy balance, glucose homeostasis, and long-term regulation of body weight [15,16,17,18,19]. Furthermore, ghrelin applies modulatory effects on the immune system as well as sleep and memory [20]. Moreover, ghrelin contributes to the synthesis of anti-inflammatory cytokines while it decreases the expression of proinflammatory cytokines [21,22,23,24].

Ghrelin was initially described as a natural peptide hormone, which is predominantly produced by gastric enteroendocrine X/A cells of the stomach and also by other organs like the brain (hypothalamus, hippocampus, and pituitary gland) and kidneys [15,25,26]. The effects of ghrelin are mediated by the growth hormone secretagogue receptor (GHS-R) with 7-transmembrane G protein-coupled receptor (GPCR), which exists in two forms: type 1a and 1b [27]. While the GHS-R1a is biologically active and consists of a 366-amino acid, the GHS-R1b has no affinity to ghrelin [28]. The GHS-R was detected in various organs such as the hypothalamus, pituitary, pancreas, thyroid, heart, adrenal glands, salivary glands, prostate, stomach, lymph nodes, and in many other organs [28,29,30]. Remarkably, ghrelin and its receptor was lately identified in the oral cavity such as in saliva, gingival crevicular fluid, gingiva, and periodontal cells [31,32,33,34,35]. In addition, evidence has focused on plasma ghrelin levels and has shown an increase in periodontitis patients as compared to periodontally healthy subjects [36], indicating that ghrelin may act as a potential link between periodontitis and systemic diseases/conditions. Moreover, our group has previously shown the effect of IL-1β on GHS-R in periodontal ligament (PDL) cells, human gingival fibroblasts (HGFs), and human osteoblast-like MG-63 cells. Furthermore, we have also shown that *F. nucleatum* regulates GHS-R in PDL and MG-63 cells [31,32,33]. Nevertheless, for a better understanding of the role and mechanism of the existing relationship between ghrelin and its receptor with periodontal diseases and periodontal tissues, more studies are needed. Therefore, the present study aimed to investigate the effects of bacterial infection on the regulation of GHS-R in vivo in rat periodontal tissues and in vitro in HGFs and also to evaluate the actions of ghrelin in HGFs.

## 2. Results

### 2.1. Expression of GHS-R in Rat Periodontal Tissues In Vivo

**Figure 1:**
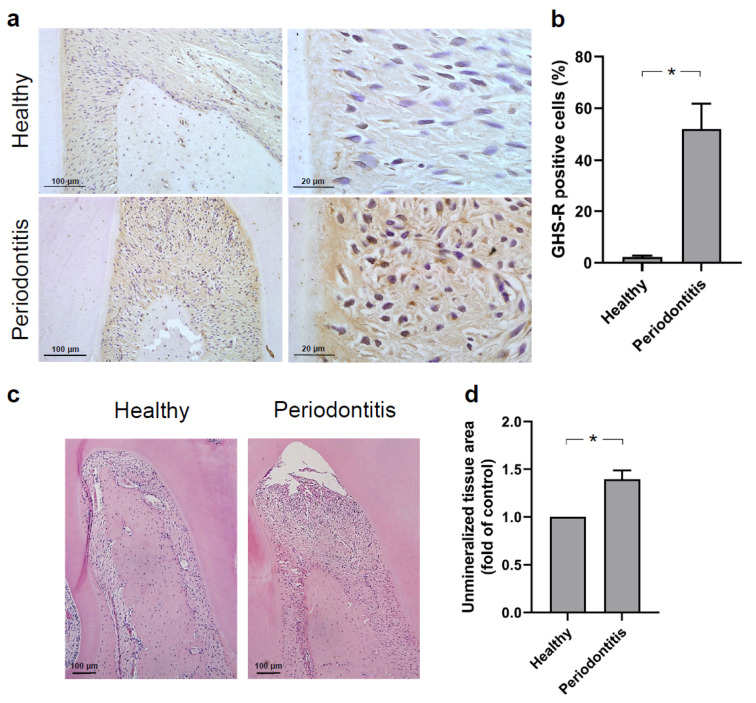
(**a**) GHS-R protein in periodontium of first molars of rats from healthy control and periodontitis groups, as evaluated by immunohistochemistry. Representative images of histological sections from one healthy control animal and one animal with periodontitis are shown. (**b**) Percentage of GHS-R positive cells is depicted. Results are expressed as mean ± SEM (n = 4 rats/group). (**c**) Representative histological sections stained with hematoxylin and eosin (H&E) and (**d**) comparison of unmineralized tissue area measured in the furcation region of first maxillary molars of rats from healthy control and periodontitis groups presented in fold change to control. Results are expressed as mean ± SEM (n = 4 rats/group). * Significant (*p* < 0.05) difference between groups.

First, the expression of GHS-R at the protein level was studied in a complex environment by using a rat ligature-induced model of experimental periodontal disease. As depicted in Figure 1a, immunohistochemistry analysis revealed that the staining against GHS-R protein was more pronounced and frequently found at periodontitis sites than at healthy control sites. The percentage of positive cells to GHS-R protein was significantly (*p* < 0.05) increased in the periodontitis group compared to the control healthy group (Figure 1b). Moreover, to confirm that the experimental periodontal disease model was functioning, bone loss was measured in the furcation region of maxillary first molars. Periodontitis was successfully achieved, as seen in Figure 1c,d. The periodontitis group showed significant (*p* < 0.05) bone loss in comparison to the healthy control group, as evidenced by an increase in unmineralized tissue area.

### 2.2. Regulation of GHS-R by F. nucleatum in HGFs In Vitro

**Figure 2:**
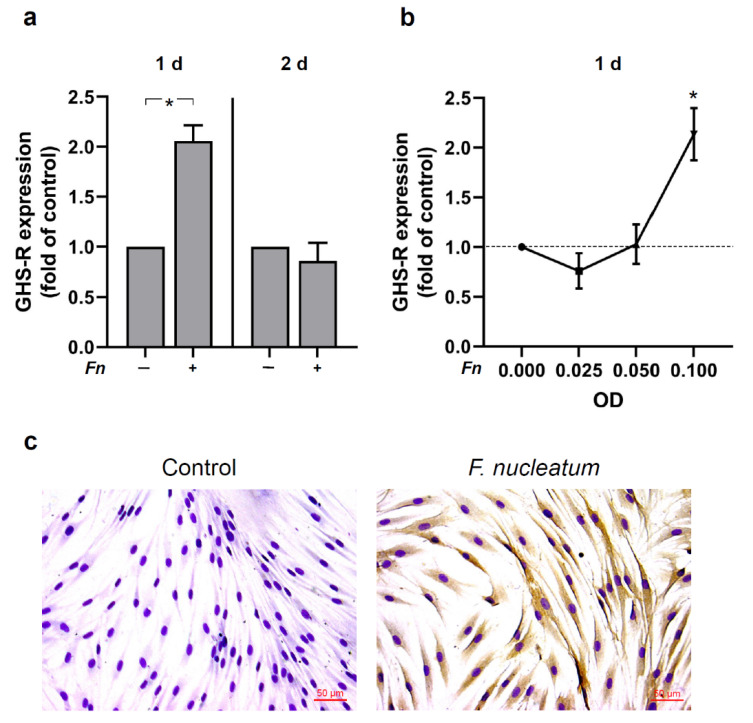
(**a**) GHS-R expression in the presence (+) and absence (−) of *F. nucleatum* (*Fn* ATCC 25586, OD_660_: 0.1) in HGF cells at 1 d and 2 d, as analyzed by real-time PCR. Mean ± SEM (n = 12). * Significant (*p* < 0.05) difference between groups. (**b**) Stimulation of GHS-R expression by various concentrations of *F. nucleatum* (*Fn* ATCC 25586, OD_660_: 0.025, 0.05, 0.1) in HGF cells at 1 d. Mean ± SEM (n = 12). * Significantly (*p* < 0.05) different from control. (**c**) GHS-R protein in the presence and absence of *F. nucleatum* (*Fn* ATCC 25586, OD_660_: 0.1) in HGF cells at 1 d, as evaluated by immunocytochemistry. Images are from one representative donor. Unstimulated cells served as control.

Next, we evaluated the regulation of GHS-R in HGFs in response to *F. nucleatum*. GHS-R was identified in HGFs and shown to be regulated by *F. nucleatum* as analyzed by real-time PCR and immunocytochemistry. As presented in Figure 2a, *F. nucleatum* significantly (*p* < 0.05) upregulated the expression of GHS-R at 1 d, but not at 2 d. Moreover, additional experiments demonstrated that the modulatory actions of *F. nucleatum* on the GHS-R expression were dose-dependent. The peak upregulation of GHS-R occurred with the highest optical density (OD) concentration of 0.1 (Figure 2b). The stimulatory effect of *F. nucleatum* on the GHS-R expression was also observed at protein level in HGFs by immunocytochemistry analysis. Parallel to the gene expression data, an increased immunoreaction for GHS-R protein was found in *F. nucleatum*-stimulated HGFs as compared to untreated control cells at 1 d (Figure 2c).

### 2.3. Signaling Pathways Involved in the Regulation of GHS-R by F. nucleatum in HGFs In Vitro

**Figure 3:**
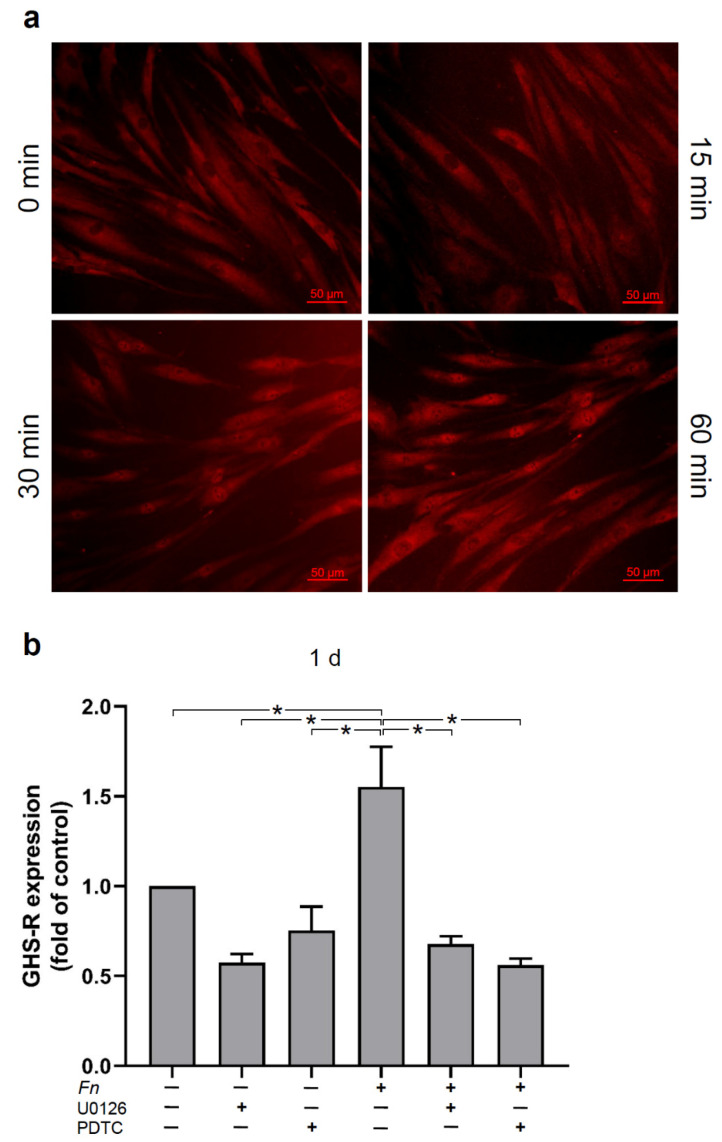
(**a**) Stimulation of NF-κB (p65) nuclear translocation by *F. nucleatum* (*Fn* ATCC 25586, OD_660_: 0.1) in HGF cells over time, as evaluated by immunofluorescence. Images are from one representative donor. (**b**) Stimulation of GHS-R expression in the presence (+) and absence (−) of *F. nucleatum* (*Fn* ATCC 25586, OD_660_: 0.1) in the presence (+) and absence (−) of U0126, a MEK1/2 inhibitor (10 µM) or PDTC, a NF-κB inhibitor (10 µM), in HGF cells at 1 d, as analyzed by real-time PCR. Mean ± SEM (n = 6). * Significant (*p* < 0.05) difference between groups. Unstimulated cells served as control.

Then, we sought to unravel signaling pathways involved in the effects of *F. nucleatum* on GHS-R in HGFs. As anticipated, *F. nucleatum*-treated HGFs triggered the NF-κB signaling pathway and caused p65 nuclear translocation at 30 min. A maximal p65 nuclear translocation occurred at 60 min, as shown by immunofluorescence microscopy (Figure 3a). When the cells were pre-incubated with U0126, a specific inhibitor of MEK1/2, or PDTC, a specific inhibitor of NF-κB signaling, the stimulatory effect of *F. nucleatum* was significantly abrogated at 1 d (Figure 3b).

### 2.4. Effects of Ghrelin on the Expressions of Proinflammatory and Chemotactic Cytokines in HGFs In Vitro

**Figure 4:**
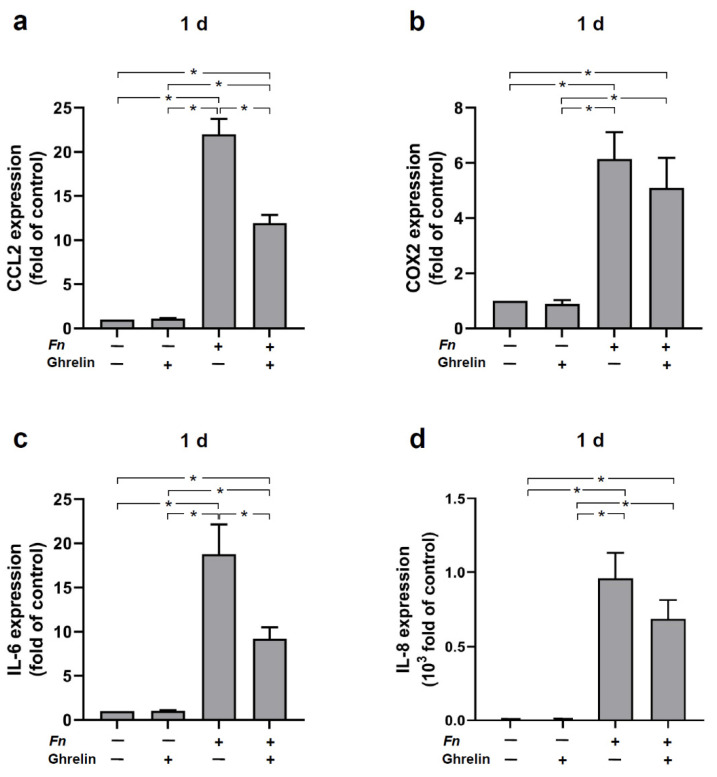
Expression of CCL2 (**a**), COX2 (**b**), IL-6 (**c**), and IL-8 (**d**) in the presence (+) and absence (−) of *F. nucleatum* (*Fn* ATCC 25586, OD_660_: 0.1) and/or ghrelin (20 nM) in HGF cells at 1 d, as determined by real-time PCR. Mean ± SEM (n = 9). * Significant (*p* < 0.05) difference between groups. Unstimulated cells served as control.

To investigate whether ghrelin would have anti-inflammatory effects, we studied the actions of ghrelin on HGFs in the presence and absence of *F. nucleatum*. As expected, *F. nucleatum* caused a significant increase of CCL2, COX2, IL-6, and IL-8 expressions in HGFs at 1 d (Figure 4a–d). However, when the cells were previously treated with ghrelin, the stimulatory effects of *F. nucleatum* were reduced for all four inflammatory mediators, with a significant inhibition for CCL2 and IL-6, as demonstrated in Figure 4a,c. Furthermore, when HGFs were treated with ghrelin alone, no significant change occurred on the cytokines expressions as compared to *F. nucleatum*-stimulated cells at 1 d (Figure 4a–d).

### 2.5. Effects of Ghrelin and F. nucleatum on Wound Healing, Proliferation, and Viability of HGFs In Vitro

**Figure 5:**
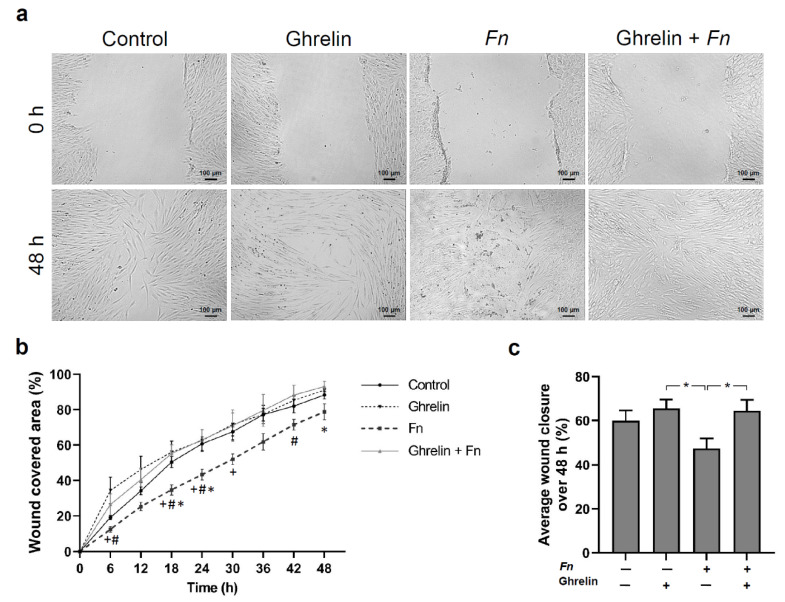
(**a**) Representative images of cell coverage from one donor at 0 and 48 h. (**b**) Percentage of cell coverage area in scratched HGF monolayers in the presence or absence of *F. nucleatum* (*Fn* ATCC 25586, OD_660_: 0.1) and/or ghrelin (20 nM) over 48 h. Mean ± SEM (*n* = 6). + Significantly different from control, # Significantly different from ghrelin, * Significantly different from ghrelin + Fn. (**c**) Percentage of average wound closure of scratched HGF monolayers shown in **b** in the presence (+) or absence (−) of *F. nucleatum* (*Fn* ATCC 25586, OD_660_: 0.1) and/or ghrelin (20 nM). Mean ± SEM. * Significant (*p* < 0.05) difference between groups. Unstimulated cells served as control.

Finally, we investigated the effect of ghrelin on wound healing of HGFs in the presence or not of *F. nucleatum*. After in vitro scratching, wounded HGF monolayers were evaluated for 48 h. Representative images illustrate the wound healing in different stimulated HGFs (Figure 5a). The wound closure rate was measured at each 6 h. *F. nucleatum* caused a decrease in the percentage of wound closure compared to all the other groups at most time points evaluated. Immediately after treatment of the wounded cells, ghrelin showed greater proliferation and migration potential with a significant (*p* < 0.05) increase in the percentage of wound covered area compared to *F. nucleatum* at 6 h, 18 h, 24 h, and 42 h (Figure 5b). Interestingly, the positive effects of ghrelin outweighed the negative effects of *F. nucleatum* as observed in the combined ghrelin and *F. nucleatum* group. The increase in wound healing by the combined group compared to *F. nucleatum* alone was significant (*p* < 0.05) at 18 h, 24 h, and 48 h (Figure 5b). *F. nucleatum* caused a significant (*p* < 0.05) reduction in the percentage of average wound closure compared to ghrelin and the combined ghrelin and *F. nucleatum* group (Figure 5c).

**Figure 6:**
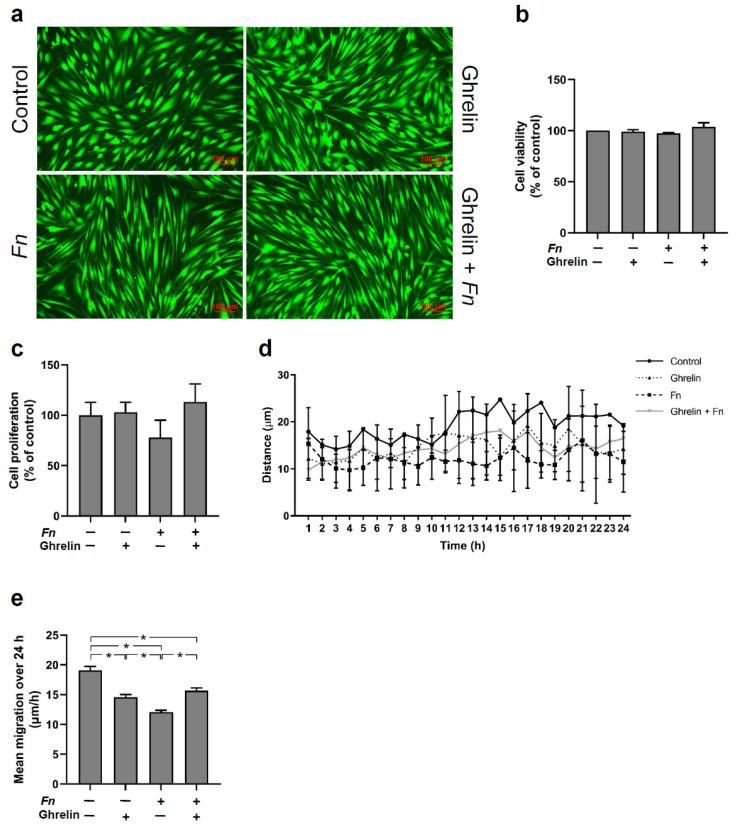
(**a**) Viability of HGFs in the presence or absence of *F. nucleatum* (*Fn* ATCC 25586; OD_660_: 0.1) and/or ghrelin (20 nM) at 2 d. Images are from one representative donor. (**b**) Percentage of cell viability in the presence (+) or absence (−) of *F. nucleatum* (*Fn* ATCC 25586; OD_660_: 0.1) and/or ghrelin (20 nM) at 2 d. Mean ± SEM (n = 6). (**c**) Percentage of cell proliferation in the presence (+) or absence (−) of *F. nucleatum* (*Fn* ATCC 25586; OD_660_: 0.1) and/or ghrelin (20 nM) at 2 d. Mean ± SEM (n = 6). (**d**) Migration of HGFs in the presence or absence of *F. nucleatum* (*Fn* ATCC 25586; OD_660_: 0.1) and/or ghrelin (20 nM) at 2 d (n = 18) measured per hour during 24 h. (**e**) Mean migration of HGFs shown in **d** in the presence (+) or absence (−) of *F. nucleatum* (*Fn* ATCC 25586; OD_660_: 0.1) and/or ghrelin (20 nM) over 24 h. Mean ± SEM. * Significant (*p* < 0.05) difference between groups. Unstimulated cells served as control.

In order to examine the negative effect of *F. nucleatum* on wound closure, cell viability, proliferation, and migration were studied. The effects of ghrelin in the presence and absence of *F. nucleatum* on HGF viability were assessed. Interestingly, there was no visible change in cell morphology following treatment with ghrelin in the presence or not of *F. nucleatum*. *F. nucleatum* alone was also not cytotoxic to the cells. HGFs remained highly viable regardless of the treatment, as depicted in Figure 6a,b. In relation to proliferation of HGFs, *F. nucleatum* induced a reduction, although not significant, as compared to the other groups, while the other groups did not show a significant difference in the regulation of cell proliferation (Figure 6c). The cell migration was measured in wounded HGF monolayers in the presence or absence of ghrelin and *F. nucleatum*. HGFs that moved the furthest into the cell-free area were monitored each hour over 24 h. Ghrelin and the combination of ghrelin and *F. nucleatum* resulted in enhanced migration as compared to *F. nucleatum* alone (Figure 6d). The mean migration was measured over 24 h. *F. nucleatum* caused a significant decrease in the mean migration of HGFs as compared to all the other groups (Figure 6e). Ghrelin and the combination of ghrelin and *F. nucleatum* also significantly reduced the mean cell migration (Figure 6e) in comparison to the unstimulated control cells.

## 3. Discussion

The present study provides original evidence that GHS-R is upregulated by bacterial infection in rat periodontal tissues in vivo and in HGFs in vitro. Furthermore, our results show that ghrelin reduces or even inhibits the proinflammatory actions of *F. nucleatum* in HGFs without interfering with cell viability and proliferation. Moreover, our experiments demonstrated that *F. nucleatum* decreases HGF wound closure while ghrelin accelerates HGF wound closure, showing that ghrelin may play a critical role in periodontal healing. Our findings suggest that ghrelin and its receptor may act as important protective molecules during bacterial infection on HGFs by increasing GHS-R and favoring wound healing through its anti-inflammatory and migration potential actions.

Ghrelin is a natural peptide hormone secreted mainly by the endocrine cells of the stomach, and its actions are intermediated by its functional receptor GHS-R [15,27]. It plays an essential role in a series of biological processes, such as regulation of appetite level, food intake, energy balance, and long-term body weight [15,16,17,18,19]. Additionally, anti-inflammatory effects of ghrelin have also been described [21,22,23,24]. Ghrelin has also been shown to accelerate the healing of oral ulcers in rats [37]. Interestingly, ghrelin and its receptor have been identified in saliva, gingival crevicular fluid, gingiva, oral epithelial cells, and periodontal fibroblasts [31,32,33,34,35]. In relation to periodontal disease, it has been demonstrated that ghrelin levels in gingival crevicular fluid were lower in periodontitis patients than in periodontally healthy patients. In contrast, when periodontitis patients were also affected by type 2 diabetes, ghrelin levels were increased in periodontitis patients [35]. Moreover, total ghrelin levels in gingival crevicular fluid from overweight/obese patients afflicted with periodontitis were lower than those from normal-weight periodontitis subjects [34]. Curiously, an increase in ghrelin plasma levels was detected in periodontitis patients as compared to periodontally healthy subjects [36]. Thus, these studies suggest a role between ghrelin and its receptor in periodontal disease.

To investigate the presence of GHS-R in periodontal tissues during periodontal disease, an experimental periodontitis model was induced by ligature. The cotton thread ligatures led to plaque accumulation around the maxillary first molars, which consequently resulted in bone loss. According to the immunohistochemistry, GHS-R was significantly increased in periodontal sites as compared to healthy control sites, suggesting that at least for the time point evaluated in our study, an increased GHS-R level is found in periodontal tissues. Similar regulatory effects of periodontitis on GHS-R expression in gingiva were observed in our previous in vivo study [33].

To better understand the occurrence of GHS-R and the effects of ghrelin on HGFs during bacterial infection, an in vitro study was performed. Interestingly, GHS-R is initially upregulated by *F. nucleatum* at 1 d; however, a decrease follows by continuous exposure of HGFs to *F. nucleatum*. The regulatory effects of *F. nucleatum* regarding the GHS-R expression were dose dependent and also detected at protein levels by immunocytochemistry. These results are in accordance with our previous results in PDL cells [33]. In order to unravel the intracellular mechanisms of *F. nucleatum*, which could possibly regulate GHS-R in HGFs, cells were pre-incubated or not with PDTC, a specific inhibitor of NF-κB signaling, or with U0126, a specific inhibitor of MAPK signaling pathways. Interestingly, we observed a significant decrease in GHS-R expression in *F. nucleatum*-stimulated cells when the cells were pre-incubated with these inhibitors, showing that *F. nucleatum*-induced upregulation of GHS-R in HGF cells was dependent on MEK1/2 and NF-κB signaling. Moreover, *F. nucleatum* induced the NF-κB signaling pathway in HGFs by p65 nuclear translocation, which was expected and is paralleled to previous reports in HGF and in periodontal cells [33,38].

In addition, we examined the regulatory effect of ghrelin on proinflammatory mediators in the presence and absence of *F. nucleatum*. Our results revealed that *F. nucleatum* induced upregulation of CCL2, COX2, IL-6, and IL-8 expressions. Higher levels of these proinflammatory cytokines/chemokines are encountered at periodontitis sites as compared to periodontally healthy sites. An increase in these markers by *F. nucleatum* has been demonstrated in PDL cells and in HGFs [38,39]. Interestingly, ghrelin significantly reduced this stimulatory effect of *F. nucleatum* and even abrogated the expressions of CCL2 and IL-6, showing an anti-inflammatory action of ghrelin. This anti-inflammatory effect of ghrelin was also previously shown by our group in PDL and MG-63 cells [31,32,33]. Other studies have shown an anti-inflammatory role of ghrelin. Administration of ghrelin, for example, reduced the IL-6 levels in mouse serum and also inhibited angiotension II-induced expression of IL-8 in human umbilical vein endothelial cells [40,41]. Furthermore, ghrelin prevented the expression of proinflammatory cytokines such as IL-1β, IL-6, and TNF- α in human T lymphocytes and monocytes [24]. Ghrelin has been shown to control inflammatory responses by inhibiting NF-κB and NLRP3 inflammasome signaling pathways [42,43]. Consequently, the expression of proinflammatory cytokines is decreased or even inhibited. These findings support the anti-inflammatory effect of ghrelin and its receptor.

Ghrelin and *F. nucleatum* alone or in combination regulated the in vitro wound healing of HGFs. Ghrelin stimulated wound closure and could improve the negative effect of *F. nucleatum* on wound fill percentage. Evidence has shown a positive effect of ghrelin on in vitro wound healing [44]. Moreover, ghrelin promoted cutaneous wound healing in mice and in rats submitted to radiation and wound injury [45,46]. Ghrelin has been shown to enhance wound healing, possibly due to the decrease in phosphorylation of MAPK p38, JNK, and NF-κB p65 [45]. Therefore, it is conceivable that the anti-inflammatory effects of ghrelin and its stimulatory actions on wound healing, as observed in our study, are mediated via inhibition of NF-κB or other signaling pathways associated with inflammation.

In order to understand whether the inhibitory action of *F. nucleatum* on wound healing was due to cell apoptosis, proliferation, and/or migration, these cell functions were investigated. Interestingly, neither ghrelin nor *F. nucleatum* exhibited significant regulation of cell viability, which implies that no stimulation was cytotoxic. Regarding cell proliferation, only *F. nucleatum* showed a trend to decrease HGF proliferation, while ghrelin and the combination of ghrelin and *F. nucleatum* did not cause a significant regulation. In our experiments, *F. nucleatum* led to a statistically significant inhibition of the mean cell migration. Interestingly, ghrelin also led to inhibition of migration, albeit to a lesser extent. As Figure 6e shows, the migration level in the combined group was higher than in the *F. nucleatum* group and about the same as in the ghrelin group. This could mean that ghrelin and *F. nucleatum* use different mechanisms to inhibit cell migration. It would be conceivable that *F. nucleatum* uses NF-κB for its inhibitory effect. It is known that ghrelin can inhibit NF-κB signal transduction and thus exert anti-inflammatory effects. Therefore, ghrelin in the combined group may have abrogated the inhibitory effect of *F. nucleatum* on cell migration by inhibiting NK-κB signal transduction. However, the inhibitory effect of ghrelin itself on cell migration was still present in the combined group because it may have been caused by a different mechanism. This could also very well explain why the inhibition of cell migration in the combined group was approximately the same as in the ghrelin group. Future studies should investigate the intracellular interactions of ghrelin and periodontal pathogenic bacteria on cell migration.

*F. nucleatum* was used in our in vitro experiments to mimic a periodontal infection in HGFs. This gram-negative anaerobic microorganism acts as a bridge bacterium between early and late colonizers in biofilm during plaque accumulation. *F. nucleatum* is associated with gingivitis as well as with periodontitis, which makes its use in HGFs reasonable. In addition, *F. nucleatum* can invade epithelial cells, fibroblasts, and PDL cells and supports other periodontal pathogens to invade host cells and to migrate to periodontal infectious sites [47,48,49]. Moreover, biofilm is composed of a complex multispecies of bacteria that are associated with periodontitis and its initiation and progression. Other periodontal pathogens used in our previous study, such as *Porphyromonas gingivalis*, *Tannerella forsythia*, *Treponema denticola*, and *Aggregatibacter actinomcycetemcomitans*, have caused upregulation of GHS-R in PDL cells [33]. In our study, we used an ultrasonicated suspension of *F. nucleatum*, which could also possibly contain different bacterial components contributing to its stimulatory effects, such as LPS or other virulence factors. Therefore, additional studies are needed to evaluate the effect of a complex bacterial biofilm on the regulation of ghrelin and its receptor in HGFs.

Within the periodontium exists various cell types, such as gingival cells, periodontal ligament cells, macrophages, osteoblasts, cementoblasts, osteoclasts, and odontoclasts. Gingival fibroblasts are important cells of the gingival connective tissue and are most frequently found in the periodontium together with the periodontal ligament fibroblasts. The other cells are also important and play a role during periodontitis. Our previous studies have demonstrated that human PDL cells and human osteoblasts also regulate GHS-R expression when exposed to *F. nucleatum* [31,32,33]. Furthermore, ghrelin and its receptor have been detected in human oral epithelial cells [50].

## 4. Materials and Methods

### 4.1. Experimental Periodontal Disease Model

A rat model was used to study the expression of GHS-R in periodontal tissues. The protocol for the animal experiment was approved by the Ethical Committee on Animal Experimentation (23/2012) at the School of Dentistry at Araraquara, São Paulo State University—UNESP. A total of eight male adult Holtzman rats with an average weight of 300 g was used and randomly distributed into two groups: healthy control group, and periodontitis group. A ligature-induced experimental periodontitis model was used as previously described [51]. In summary, after general anesthesia was applied (0.08 mL of 10% ketamine hydrochloride and 0.04 mL of 2% xylazine hydrochloride per 100 g of body weight), cotton ligatures were inserted around the cervical area of the maxillary first molars bilaterally. The ligatures were kept in place during the entire experimental period. After 12 d, animals were euthanized by anesthetic overdose. The maxillary jaws were fixed in 4% paraformaldehyde for 48 h, decalcified in EDTA (10%, 0.5 M) for 3 months, and then embedded in paraffin. Subsequently, serial parasagittal sections of five μm were mounted on slides for immunohistochemistry and histomorphometric analyses.

### 4.2. Immunohistochemistry Analysis

Immunohistochemical analysis to evaluate ghrelin levels within the periodontal ligament was performed by the avidin-biotin-peroxidase (ABC) method using the LSAB kit (Dako, Glostrup, Denmark), according to the manufacturer’s instructions. Silanized slides (Dako) were mounted with serial parasagittal sections of five-µm thickness. Antigen retrieval was carried out by hitting cuts in the Rodent Decloaker reagent (Biocare Medical; Concord, CA, USA) in a Decloaking Chamber (Biocare Medical) at 80 °C for 30 min. Endogenous peroxidase activity was blocked with a 5% hydrogen peroxide solution. Nonspecific binding blocking was performed in 5% bovine serum albumin (BSA, Sigma-Aldrich, Munich, Germany) in phosphate-buffered saline (PBS, Invitrogen, Karlsruhe, Germany). The sections were incubated with rabbit polyclonal antibody anti-ghrelin receptor (1:100, ab85104, Abcam, Cambridge, England). Negative control sections were incubated with 1% PBS to assess unspecific background staining overnight at 4 °C. Tissue sections were washed and incubated with biotinylated immunoglobulin (Dako) at room temperature (RT) for 45 min and subsequently washed again and incubated with avidin-biotin-peroxidase complex (Dako) at RT for 45 min. Diaminobenzidine (DAB, Dako) was used as a chromogen substrate. All sections were counterstained with Carrazi’s hematoxylin. In each section, 6 images were taken using optical microscopy (LEICA microsystem GmbH, Wetzlar, Germany) at 20× and 100× magnification. Positive cells were counted in a rectangular region of interest (ROI) measuring 66,074 µm^2^ by a blinded and calibrated examiner using the public domain software, ImageJ 1.53 (National Institutes of Health, NIH, Bethesda, MD, USA), for processing and analyzing scientific images [52].

### 4.3. Histomorphometric Analysis

Morphometric changes were assessed on slides stained with hematoxylin and eosin (H&E). Images from the histological sections were taken using a light microscope (LEICA) under 4× magnification and a Leica DFC 300 FX digital camera. A total of three sections was selected from each tooth from four animals in each group. The area of unmineralized tissue in the furcation region was determined by histomorphometric analysis as previously described [53]. A ROI comprised of a 1000-μm zone under the furcation roof of the maxillary first molar was studied. In order to calculate the area of unmineralized tissue, the total area of the ROI was measured and the area of mineralized bone tissue was subtracted. Then, this measurement was expressed as fold-change compared to the control. A single, blinded, and calibrated examiner performed the histomorphometric analysis using ImageJ analysis system.

### 4.4. Cell Culture and Treatment

HGFs were obtained from six healthy gingiva individuals (mean age: 21.0 ± 1.6 years, min–max: 16–31 years, gender: 4 male/2 female) submitted to wisdom tooth extraction in the Department of Oral Surgery of the University of Bonn. Written informed consent and approval of the Ethics Committee of the University of Bonn were obtained. Cells were maintained in Dulbecco’s modified Eagle’s medium (DMEM, Invitrogen) supplemented with 10% fetal bovine serum (FBS, Invitrogen), 100 units penicillin, and 100 μg/mL streptomycin (Invitrogen) at 37 °C in a humidified atmosphere of 5% CO_2_. All experiments were carried out with cells from the third to the fifth passage. Approximately 50,000 cells per well were seeded on tissue culture plates (Corning, Corning, NY, USA) until they reached 80% confluence. Every other day the culture medium was changed. Cells were serum-deprived in medium containing 0.1% FBS one day before the start of the experiments. In order to mimic microbial infections in vitro, cells were stimulated with the inactivated oral pathogenic bacteria *Fusobacterium nucleatum* ATCC 25586 (optical density (OD): 0.025, 0.05, and 0.1). The bacteria strain was pre-cultivated on Schaedler agar plates (Oxoid, Basingstoke, UK) in an anaerobic atmosphere for 48 h. Thereafter, bacteria were suspended in PBS (OD_660nm_ = 1, equivalent to 1.2 × 10^9^ bacterial cells/mL) and then exposed twice to ultrasonication (160 W for 15 min), resulting in complete death. In order to investigate the possible anti-inflammatory effects of ghrelin on HGFs, cells were pre-incubated with ghrelin (20 nM, human n-octanoylated ghrelin, Pepta Nova, Sandhausen, Germany) 45 min prior to the treatment of cells with *F. nucleatum*. Ghrelin was also used to treat HGFs alone to study its modulatory effect on the expression of proinflammatory mediators. In addition, HGFs were pre-incubated with pyrrolidine dithiocarbamate (PDTC; 10 µM; Calbiochem, San Diego, CA, USA), a specific inhibitor of NF-κB signaling, and U0126 (10 µM; Calbiochem), a specific inhibitor of MEK1/2, two important components of the MAPK signaling pathway, in order to elucidate the intracellular signaling mechanisms exploited by *F. nucleatum* to modulate GHS-R expression. The inhibitors were added to the cells 1 h before the stimulation with *F. nucleatum*. Finally, the effects of ghrelin in the presence or not of *F. nucleatum* were investigated on wound healing, viability, and proliferation of HGFs.

### 4.5. Real-Time PCR

Total RNA from HGFs was isolated using an RNeasy Mini Kit (Qiagen, Hilden, Germany) according to the manufacturer’s protocol. RNA concentration was calculated by using the spectrophotometer NanoDrop ND-2000 (Thermo Fisher Scientific, Wilmington, NC, USA). A total of 1 µg of RNA was reverse transcribed using the iScript™ Select Synthesis Kit (Bio-Rad Laboratories, Munich, Germany) at 42 °C for 90 min followed by 85 °C for 5 min, as per the manufacturer’s instructions. Expressions of GHS-R, CCL2, COX2, IL-6, IL-8, and glyceraldehyde-3-phosphate dehydrogenase (GAPDH) as a reference control gene were detected by quantitative RT-PCR using the iCycler iQ™ Real-Time PCR Detection System (Bio-Rad), SYBR Green (SsoAdvanced™ Universal SYBR^®^ Green Supermix, Bio-Rad), and specific primers (QuantiTect Primer Assay, Qiagen). Amplification occurred as follows: initial heat at 95 °C for 5 min, followed by 40 cycles of denaturation at 95 °C for 10 s, and combined annealing/extension at 60 °C for 30 s. Data analysis was applied using the comparative threshold cycle method.

### 4.6. Immunocytochemistry

For the detection of GHS-R, HGFs were grown on plastic coverslips (Thermo Fisher Scientific, Darmstadt, Germany) of 13 mm diameter in 24-well plates in the presence or absence of *F. nucleatum* for 1 d. After that, the cell monolayers were fixed in 4% paraformaldehyde (Sigma-Aldrich) at pH 7.4 and RT for 10 min. Subsequently, cells were permeabilized in 0.1% Triton X-100 (Sigma-Aldrich) for 5 min and then blocked using serum block (Dako) for 20 min. Afterwards, cells were incubated with rabbit polyclonal primary antibody to GHS-R (1:500, Abcam) for 90 min. Next, cells were incubated with goat anti-rabbit IgG HRP secondary antibody (Dako) for 45 min. Then, DAB solution, which was freshly prepared (3,3′-diaminobenzidine substrate diluted 1:10 in peroxidase substrate buffer), was added to the cells and left for 5–10 min at RT in dark. Cell monolayers were washed with PBS after each step. Cells were counterstained in Mayer’s hematoxylin solution for 5 s and washed thoroughly with water. Finally, HGFs were mounted with DePeX (SERVA Electrophoresis, Heidelberg, Germany). The slides were examined using an Axioskop 2 microscope (Zeiss, Oberkochen, Germany) equipped with a 20× objective. Images were captured using an AxioCam MRc microscope camera (Carl Zeiss) and the AxioVision 4.7 software (Carl Zeiss). Untreated cells served as control.

### 4.7. Immunofluorescence

HGFs were cultured on plastic coverslips in the presence or absence of *F. nucleatum* for 90 min. Then, the cells were fixed and permeabilized as described above. Afterwards, the cell monolayers were blocked with nonfat dry milk (Bio-Rad) for 1 h and then incubated with a rabbit anti-nuclear factor-κB p65 (E498) primary antibody (1:100, Cell Signaling Technology, Danvers, MA, USA) at RT for 90 min. Subsequently, the cells were incubated with CY3-conjugated goat anti-rabbit IgG secondary antibody (1:1000, Abcam) at RT for 45 min. After each step, cells were washed with PBS. Finally, the cells were observed by using a ZOE™ fluorescent cell imager (Bio-Rad) with a 20× objective. An integrated digital 5MP CMOS camera was used to capture the images. Untreated cells were used as a control.

### 4.8. In Vitro Wound Healing, Cell Viability, Proliferation, and Migration

In order to evaluate the effects of ghrelin and *F. nucleatum* on wound healing of HGFs, a well-established in vitro wound healing model was used according to previous studies [54,55]. HGFs were cultivated in 35 mm culture dishes (Thermo Fisher) and grown to 100% confluence. One day after reducing the FBS concentration, a wound was created in a standardized manner using a sterile 100 uL pipette tip, leading to cell-free areas in the cell monolayers. Then, a number of washing steps with PBS and DMEM was performed to remove all non-adherent cells. After that, the cells were cultured in the presence and absence of ghrelin and *F. nucleatum* alone or in combination for 2 d. Wound closure was monitored over time using JuLI™ Br and JuLI™ Br PC software (both NanoEnTek, Seoul, Korea). The images were analyzed by a blinded and calibrated examiner, using the ImageJ analysis system. The wound closure area was calculated and transformed in percentage.

Additionally, in order to evaluate cell viability, the LIVE/DEAD Viability/Cytotoxicity Kit (Invitrogen) assay was used. After 48 h, the medium was removed, and the cell monolayers were rinsed twice with PBS and incubated with the ethidium-calcein solution at RT for 30 min. Next, cells were washed and left with PBS. Then, fluorescence microscopy was performed by using a ZOE™ fluorescent cell imager. Untreated cells were used as a control.

Cell proliferation was performed using 0.4% trypan blue solution (BioWhittaker, Walkersville, MD, USA). The numbers of stained and non-stained cells were determined by using an automated cell counter (Luna, Logos Biosystems, Gyeonggi-do, Korea).

Migration of HGFs was analyzed by examining six cells from each donor that moved the furthest into the cell-free area per hour over 24 h. The cells were marked and followed for 24 h. Subsequently, all images were transferred to the ImageJ analysis system to measure the distance of movement per hour.

### 4.9. Statistical Analysis

The GraphPad Prism 9 (GraphPad Software Inc., San Diego, CA, USA) software was used for the data analysis. For quantitative analysis, mean values and standard errors of the mean (SEM) were calculated. Differences between groups were considered significant at *p* < 0.05. Parametric (t-test and ANOVA followed by the post hoc Tukey’s test) and non-parametric tests (Wilcoxon and Mann–Whitney-U tests) were applied for statistical analysis.

## 5. Conclusions

Our study yields original evidence that GHS-R expression is enhanced by bacterial infection in rat periodontal tissues in vivo and in HGFs in vitro. Moreover, our findings show that ghrelin reduces or even inhibits the proinflammatory actions of *F. nucleatum* in HGFs without affecting cell viability and proliferation. Furthermore, our experiments revealed that *F. nucleatum* decreases HGF wound closure while ghrelin accelerates, indicating that ghrelin may play a critical role in periodontal healing. Our findings suggest that ghrelin and its receptor may act as important protective molecules during bacterial infection in HGFs by increasing GHS-R and favoring wound healing through its anti-inflammatory and migration potential actions.

## References

[1] Nocini R., Lippi G., Mattiuzzi C. (2020). Periodontal disease: The portrait of an epidemic. J. Public Health Emerg..

[2] Papapanou P.N., Sanz M., Buduneli N., Dietrich T., Feres M., Fine D.H., Flemmig T.F., Garcia R., Giannobile W.V., Graziani F. (2018). Periodontitis: Consensus report of workgroup 2 of the 2017 World Workshop on the Classification of Periodontal and Peri-Implant Diseases and Conditions. J. Clin. Periodontol..

[3] Beikler T., Flemmig T.F. (2011). Oral biofilm-associated diseases: Trends and implications for quality of life, systemic health and expenditures. Periodontology 2000.

[4] Botelho J., Machado V., Leira Y., Proenca L., Chambrone L., Mendes J.J. (2021). Economic burden of periodontitis in the United States and Europe—An updated estimation. J. Periodontol..

[5] Pihlstrom B.L., Michalowicz B.S., Johnson N.W. (2005). Periodontal diseases. Lancet.

[6] Sbordone L., Bortolaia C. (2003). Oral microbial biofilms and plaque-related diseases: Microbial communities and their role in the shift from oral health to disease. Clin. Oral Investig..

[7] Yucel-Lindberg T., Bage T. (2013). Inflammatory mediators in the pathogenesis of periodontitis. Expert Rev. Mol. Med..

[8] Tatakis D.N., Kumar P.S. (2005). Etiology and pathogenesis of periodontal diseases. Dent. Clin. N. Am..

[9] Romandini M., Baima G., Antonoglou G., Bueno J., Figuero E., Sanz M. (2021). Periodontitis, Edentulism, and Risk of Mortality: A Systematic Review with Meta-analyses. J. Dent. Res..

[10] Stohr J., Barbaresko J., Neuenschwander M., Schlesinger S. (2021). Bidirectional association between periodontal disease and diabetes mellitus: A systematic review and meta-analysis of cohort studies. Sci. Rep..

[11] Gobin R., Tian D., Liu Q., Wang J. (2020). Periodontal Diseases and the Risk of Metabolic Syndrome: An Updated Systematic Review and Meta-Analysis. Front. Endocrinol..

[12] Martinez-Herrera M., Silvestre-Rangil J., Silvestre F.J. (2017). Association between obesity and periodontal disease. A systematic review of epidemiological studies and controlled clinical trials. Med. Oral Patol. Oral Cir. Bucal..

[13] Gaio E.J., Haas A.N., Rosing C.K., Oppermann R.V., Albandar J.M., Susin C. (2016). Effect of obesity on periodontal attachment loss progression: A 5-year population-based prospective study. J. Clin. Periodontol..

[14] Moura-Grec P.G., Marsicano J.A., Carvalho C.A., Sales-Peres S.H. (2014). Obesity and periodontitis: Systematic review and meta-analysis. Cien. Saude Colet..

[15] Slotwinska S.M. (2020). Ghrelin and oral diseases. Cent. Eur. J. Immunol..

[16] Alamri B.N., Shin K., Chappe V., Anini Y. (2016). The role of ghrelin in the regulation of glucose homeostasis. Horm. Mol. Biol. Clin. Investig..

[17] Gil-Campos M., Aguilera C.M., Canete R., Gil A. (2006). Ghrelin: A hormone regulating food intake and energy homeostasis. Br. J. Nutr..

[18] Cummings D.E., Weigle D.S., Frayo R.S., Breen P.A., Ma M.K., Dellinger E.P., Purnell J.Q. (2002). Plasma ghrelin levels after diet-induced weight loss or gastric bypass surgery. N. Engl. J. Med..

[19] Horvath T.L., Diano S., Sotonyi P., Heiman M., Tschop M. (2001). Minireview: Ghrelin and the regulation of energy balance--a hypothalamic perspective. Endocrinology.

[20] Gahete M.D., Rincon-Fernandez D., Villa-Osaba A., Hormaechea-Agulla D., Ibanez-Costa A., Martinez-Fuentes A.J., Gracia-Navarro F., Castano J.P., Luque R.M. (2014). Ghrelin gene products, receptors, and GOAT enzyme: Biological and pathophysiological insight. J. Endocrinol..

[21] Khatib M.N., Shankar A.H., Kirubakaran R., Gaidhane A., Gaidhane S., Simkhada P., Syed Z.Q. (2018). Ghrelin for the management of cachexia associated with cancer. Cochrane Database Syst. Rev..

[22] Chen J.A., Splenser A., Guillory B., Luo J., Mendiratta M., Belinova B., Halder T., Zhang G., Li Y.P., Garcia J.M. (2015). Ghrelin prevents tumour- and cisplatin-induced muscle wasting: Characterization of multiple mechanisms involved. J. Cachexia Sarcopenia Muscle.

[23] Waseem T., Duxbury M., Ito H., Ashley S.W., Robinson M.K. (2008). Exogenous ghrelin modulates release of pro-inflammatory and anti-inflammatory cytokines in LPS-stimulated macrophages through distinct signaling pathways. Surgery.

[24] Dixit V.D., Schaffer E.M., Pyle R.S., Collins G.D., Sakthivel S.K., Palaniappan R., Lillard J.W., Taub D.D. (2004). Ghrelin inhibits leptin- and activation-induced proinflammatory cytokine expression by human monocytes and T cells. J. Clin. Investig..

[25] Date Y., Kojima M., Hosoda H., Sawaguchi A., Mondal M.S., Suganuma T., Matsukura S., Kangawa K., Nakazato M. (2000). Ghrelin, a novel growth hormone-releasing acylated peptide, is synthesized in a distinct endocrine cell type in the gastrointestinal tracts of rats and humans. Endocrinology.

[26] Kojima M., Hosoda H., Date Y., Nakazato M., Matsuo H., Kangawa K. (1999). Ghrelin is a growth-hormone-releasing acylated peptide from stomach. Nature.

[27] Adams E.F., Huang B., Buchfelder M., Howard A., Smith R.G., Feighner S.D., van der Ploeg L.H., Bowers C.Y., Fahlbusch R. (1998). Presence of growth hormone secretagogue receptor messenger ribonucleic acid in human pituitary tumors and rat GH3 cells. J. Clin. Endocrinol. Metab..

[28] Howard A.D., Feighner S.D., Cully D.F., Arena J.P., Liberator P.A., Rosenblum C.I., Hamelin M., Hreniuk D.L., Palyha O.C., Anderson J. (1996). A receptor in pituitary and hypothalamus that functions in growth hormone release. Science.

[29] Gnanapavan S., Kola B., Bustin S.A., Morris D.G., McGee P., Fairclough P., Bhattacharya S., Carpenter R., Grossman A.B., Korbonits M. (2002). The tissue distribution of the mRNA of ghrelin and subtypes of its receptor, GHS-R, in humans. J. Clin. Endocrinol. Metab..

[30] Papotti M., Ghe C., Cassoni P., Catapano F., Deghenghi R., Ghigo E., Muccioli G. (2000). Growth hormone secretagogue binding sites in peripheral human tissues. J. Clin. Endocrinol. Metab..

[31] Nokhbehsaim M., Memmert S., Damanaki A., Nanayakkara S., Zhou X., Jager A., Deschner J. (2019). Effect of interleukin-1beta on ghrelin receptor in periodontal cells. Clin. Oral Investig..

[32] Nokhbehsaim M., Nogueira A.V.B., Memmert S., Damanaki A., Eick S., Cirelli J.A., Deschner J. (2019). Regulation of ghrelin receptor by microbial and inflammatory signals in human osteoblasts. Braz. Oral Res..

[33] Nokhbehsaim M., Damanaki A., Nogueira A.V.B., Eick S., Memmert S., Zhou X., Nanayakkara S., Gotz W., Cirelli J.A., Jager A. (2017). Regulation of Ghrelin Receptor by Periodontal Bacteria In Vitro and In Vivo. Mediat. Inflamm..

[34] Jentsch H.F.R., Arnold N., Richter V., Deschner J., Kantyka T., Eick S. (2017). Salivary, gingival crevicular fluid and serum levels of ghrelin and chemerin in patients with periodontitis and overweight. J. Periodontal. Res..

[35] Mohamed H.G., Idris S.B., Mustafa M., Ahmed M.F., Astrom A.N., Mustafa K., Ibrahim S.O. (2015). Impact of Chronic Periodontitis on Levels of Glucoregulatory Biomarkers in Gingival Crevicular Fluid of Adults with and without Type 2 Diabetes. PLoS ONE.

[36] Yilmaz G., Kirzioglu F.Y., Doguc D.K., Kocak H., Orhan H. (2014). Ghrelin levels in chronic periodontitis patients. Odontology.

[37] Warzecha Z., Kownacki P., Ceranowicz P., Dembinski M., Cieszkowski J., Dembinski A. (2013). Ghrelin accelerates the healing of oral ulcers in non-sialoadenectomized and sialoadenectomized rats. J. Physiol. Pharmacol..

[38] Kang W., Jia Z., Tang D., Zhang Z., Gao H., He K., Feng Q. (2019). Fusobacterium nucleatum Facilitates Apoptosis, ROS Generation, and Inflammatory Cytokine Production by Activating AKT/MAPK and NF-kappaB Signaling Pathways in Human Gingival Fibroblasts. Oxid. Med. Cell Longev..

[39] Rath-Deschner B., Memmert S., Damanaki A., Nokhbehsaim M., Eick S., Cirelli J.A., Gotz W., Deschner J., Jager A., Nogueira A.V.B. (2020). CXCL1, CCL2, and CCL5 modulation by microbial and biomechanical signals in periodontal cells and tissues-in vitro and in vivo studies. Clin. Oral Investig..

[40] Khazaei M., Tahergorabi Z. (2015). Serum inflammatory markers in obese mice: Effect of ghrelin. Adv. Biomed. Res..

[41] Deng B., Fang F., Yang T., Yu Z., Zhang B., Xie X. (2015). Ghrelin inhibits AngII -induced expression of TNF-alpha, IL-8, MCP-1 in human umbilical vein endothelial cells. Int. J. Clin. Exp. Med..

[42] Qu R., Chen X., Hu J., Fu Y., Peng J., Li Y., Chen J., Li P., Liu L., Cao J. (2019). Ghrelin protects against contact dermatitis and psoriasiform skin inflammation by antagonizing TNF-alpha/NF-kappaB signaling pathways. Sci. Rep..

[43] Liu F., Li Z., He X., Yu H., Feng J. (2019). Ghrelin Attenuates Neuroinflammation and Demyelination in Experimental Autoimmune Encephalomyelitis Involving NLRP3 Inflammasome Signaling Pathway and Pyroptosis. Front. Pharmacol..

[44] Li H., Zhang X., Feng L. (2021). Ghrelin Regulates Cyclooxygenase-2 Expression and Promotes Gastric Cancer Cell Progression. Comput. Math. Methods Med..

[45] Liu C., Huang J., Li H., Yang Z., Zeng Y., Liu J., Hao Y., Li R. (2016). Ghrelin accelerates wound healing through GHS-R1a-mediated MAPK-NF-kappaB/GR signaling pathways in combined radiation and burn injury in rats. Sci. Rep..

[46] Liu C., Hao Y., Huang J., Li H., Yang Z., Zeng Y., Liu J., Li R. (2017). Ghrelin accelerates wound healing in combined radiation and wound injury in mice. Exp. Dermatol..

[47] He J., Huang W., Pan Z., Cui H., Qi G., Zhou X., Chen H. (2012). Quantitative analysis of microbiota in saliva, supragingival, and subgingival plaque of Chinese adults with chronic periodontitis. Clin. Oral Investig..

[48] Signat B., Roques C., Poulet P., Duffaut D. (2011). Fusobacterium nucleatum in periodontal health and disease. Curr. Issues Mol. Biol..

[49] Dabija-Wolter G., Cimpan M.R., Costea D.E., Johannessen A.C., Sornes S., Neppelberg E., Al-Haroni M., Skaug N., Bakken V. (2009). Fusobacterium nucleatum enters normal human oral fibroblasts in vitro. J. Periodontol..

[50] Groschl M., Topf H.G., Bohlender J., Zenk J., Klussmann S., Dotsch J., Rascher W., Rauh M. (2005). Identification of ghrelin in human saliva: Production by the salivary glands and potential role in proliferation of oral keratinocytes. Clin. Chem..

[51] Nogueira A.V., de Molon R.S., Nokhbehsaim M., Deschner J., Cirelli J.A. (2017). Contribution of biomechanical forces to inflammation-induced bone resorption. J. Clin. Periodontol..

[52] Schneider C.A., Rasband W.S., Eliceiri K.W. (2012). NIH Image to ImageJ: 25 years of image analysis. Nat. Methods.

[53] Rath-Deschner B., Nogueira A.V.B., Beisel-Memmert S., Nokhbehsaim M., Eick S., Cirelli J.A., Deschner J., Jager A., Damanaki A. (2022). Interaction of periodontitis and orthodontic tooth movement-an in vitro and in vivo study. Clin. Oral Investig..

[54] Weusmann J., Deschner J., Imber J.C., Damanaki A., Leguizamon N.D.P., Nogueira A.V.B. (2021). Cellular effects of glycine and trehalose air-polishing powders on human gingival fibroblasts in vitro. Clin. Oral Investig..

[55] Memmert S., Nokhbehsaim M., Damanaki A., Nogueira A.V.B., Papadopoulou A.K., Piperi C., Basdra E.K., Rath-Deschner B., Gotz W., Cirelli J.A. (2018). Role of cathepsin S In periodontal wound healing-an in vitro study on human PDL cells. BMC Oral Health.

